# The Sequelæ of Enteric Fever—V

**Published:** 1908-07-04

**Authors:** 


### Medicine.

### THE SEQUELÆ OF ENTERIC FEVER—V.

We have discussed the majority of the sequelæ of enteric fever in the four short articles upon the subject already published; there remain a few others, however, upon each of which a few words may be said in conclusion.

### Peripheral Neuritis.

Occasionally extensive multiple neuritis, affecting both upper and lower extremities, with sensory disturbances and atrophy similar to those met with in alcoholic subjects, is seen. As a rule, however, the neuritis is irregular in its distribution, rather than symmetrical as a peripheral neuritis ought to be according to the usual definition of the term. It may be located in a plexus of nerves on one side of the body only, or on one side of the body very much more than upon the other. It may be restricted to the root or trunk of a single nerve. The trouble usually sets in with excessive pain in the deep parts of the affected limb, or along the course of the affected nerve, followed by numbness and paresis. Sometimes the pain and other sensory phenomena may be extreme without the motor functions being much involved; in other cases there may be complete paralysis of the muscles, supplied by the affected nerve or nerves, followed by marked atrophy and the reaction of degeneration. It is impossible to give names to all the different types of this neuritis; sometimes its form resembles a sciatica; or the front of the thigh may be involved by a lesion which might be designated anterior cruritis; the arms may be affected as well as, or apart from, the legs, one particularly troublesome form being that in which the parts about the shoulder joint are involved—the wasting of the muscles causes the humerus to fall away from the glenoid cavity, thus exerting additional traction upon the branches of the brachial plexus, and leading to excruciating pains.

Paresis of cranial nerves is very rare after typhoid fever, but Dr. Dreschfeld records a case in which the fifth was affected both as to its sensory and its motor roots, recovery being but partial.

The prognosis of post-typhoidal peripheral neuritis is upon the whole quite favourable, and the treatment is precisely similar to that of the same lesion due to other causes.

### Contracture of the Legs.

If in any tedious debilitating malady the position of the patient is long maintained in such a way that any of the muscles are not compelled, from time to time, to extend to their full length, there is a liability that they will become contracted and shortened. It has happened, for example, that a young patient has been ill of typhoid fever for a great many weeks—so ill that she was not expected to recover; she has been disturbed as little as possible, and she has maintained a posture in bed with her thighs drawn up towards her abdomen and her legs flexed at the knee; she has ultimately recovered, but to the dismay of all, it is found that she is now unable to straighten her legs; the latter are permanently flexed, both at the hips and at the knees; when she is strong enough, tenotomy of the hamstrings is performed, and so forth; the contractures are relieved partially, but the patient is never able to get her legs properly straight again; she is crippled by contracture of the legs.

Naturally this sequela is rare, because in most cases nowadays, the skilled nurses who attend patients wash them from head to foot each day and in doing so ensure that the legs are straightened at least once in each twenty-four hours. The possibility of this untoward sequel of the illness should never be forgotten, however; the legs should not be allowed to remain flexed or in one position for days together; if the muscles are passively elongated from time to time, the misfortune will not result. Once seen, a case of the kind will never be forgotten.

### Thrombosis.

Thrombosis might have been mentioned earlier in the list of the sequelæ of typhoid fever, because it is not very uncommon. There is, however, little to say about post typhoidal thrombosis in particular, for it does not differ greatly, either as to prognosis or as to treatment, from thrombosis due to other causes. As a rule it is of the "simple" type; that is to say, the clot does not tend to break down and lead to septic embolism of the lungs, and pyæmia. Though "simple" in this respect, it is like other "simple" thromboses in the fact that it has a microbial cause just as much as "infective" thromboses have. A good deal has been written lately upon the coagulability of the blood in typhoid fever; it has been stated that the blood is more coagulable than it should be, that this is a main cause for the thrombosis, and that the latter may be inhibited by the exhibition of such drugs as potassium citrate. This may be the case to a small extent, but there is a considerable amount of evidence to show that mere increased coagulability of the blood will not cause thrombosis unless there is a bacterial invasion of the wall of the affected vein as well. The typhoid bacillus may, perhaps, be the offending organism in some of the post-typhoidal cases, but the commonest bacterium to find in so-called "simple" thrombi, whatever disease they arise in, is the *bacillus proteus vulgaris*. Dr. Bryant and Dr. Pakes found this organism in quite a number of consecutive cases. It would seem that the bacillus proteus vulgaris, which is practically innocuous to healthy persons, becomes harmful to those who are debilitated by disease, and that amongst the lesions it produces is phlebitis and venous thrombosis. This explains the clinical facts that nearly every such case has pyrexia, which lasts for days or weeks, and that there is visible evidence of active inflammation along the course of the affected vein.

After typhoid fever, the commonest thrombosis is that of one or other internal saphenous vein or its connections. The treatment is the same as it is for non-typhoidal cases.

Spontaneous Rupture of Muscles: Zenker's Degeneration.

A rare, but very remarkable, sequela of typhoid fever is the spontaneous rupture of a muscle, or of part of a muscle, when the patient first begins to exert himself physically during convalescence. The least uncommon site for the lesion is the rectus abdominis muscle of one side or the other. It is not common for the entire muscle to snap across, but several fibres in it may give way, with sudden pain followed by some deformity in the outline of the affected muscle. Effusion into the sheath follows the rupture, but the condition is not dangerous; rest and time cure the symptoms, though it may be possible to feel a permanent irregularity of outline when the muscle is put into contraction. The cause of the lesion is a degeneration in the fibres of the muscle, first described by Zenker, and referred to as "vitreous" or "Zenker's" degeneration. The affected fibres look dull and opaque; microscopically they are seen to have lost their striation, so that the sarcolemma is filled with masses of clear, homogeneous, glass-like substance; the connective tissue cells of the muscle undergo proliferation; some authors regard the change as a coagulation-necrosis, caused by the high fever.

Orchitis.

In conclusion, one word must be said about orchitis. It is not extremely uncommon after typhoid fever, and like many others of the sequelæ that have been discussed, it may be long delayed. Its chief importance lies in the fact that it may too easily be attributed to a cause other than past enteric fever.

